# Biomarker-driven molecular imaging probes in radiotherapy

**DOI:** 10.7150/thno.97768

**Published:** 2024-07-02

**Authors:** Haonan Li, Qiyong Gong, Kui Luo

**Affiliations:** 1 Department of Radiology, Huaxi MR Research Center (HMRRC), Frontiers Science Center for Disease-Related Molecular Network, State Key Laboratory of Biotherapy, West China Hospital, Sichuan University, No. 37 Guoxue Alley, Chengdu 610041, China.; 2 Functional and Molecular Imaging Key Laboratory of Sichuan Province and Research Unit of Psychoradiology, Chinese Academy of Medical Sciences, Chengdu 610041, China.; 3 Department of Radiology, West China Xiamen Hospital of Sichuan University, 699 Jinyuan Xi Road, Jimei District, 361021 Xiamen, Fujian, China.

**Keywords:** biomarker, molecular imaging, radiotherapy, imaging probe, nanoparticle

## Abstract

**Background:** Biomarker-driven molecular imaging has emerged as an integral part of cancer precision radiotherapy. The use of molecular imaging probes, including nanoprobes, have been explored in radiotherapy imaging to precisely and noninvasively monitor spatiotemporal distribution of biomarkers, potentially revealing tumor-killing mechanisms and therapy-induced adverse effects during radiation treatment.

**Methods:** We summarized literature reports from preclinical studies and clinical trials, which cover two main parts: 1) Clinically-investigated and emerging imaging biomarkers associated with radiotherapy, and 2) instrumental roles, functions, and activatable mechanisms of molecular imaging probes in the radiotherapy workflow. In addition, reflection and future perspectives are proposed.

**Results:** Numerous imaging biomarkers have been continuously explored in decades, while few of them have been successfully validated for their correlation with radiotherapeutic outcomes and/or radiation-induced toxicities. Meanwhile, activatable molecular imaging probes towards the emerging biomarkers have exhibited to be promising in animal or small-scale human studies for precision radiotherapy.

**Conclusion:** Biomarker-driven molecular imaging probes are essential for precision radiotherapy. Despite very inspiring preliminary results, validation of imaging biomarkers and rational design strategies of probes await robust and extensive investigations. Especially, the correlation between imaging biomarkers and radiotherapeutic outcomes/toxicities should be established through multi-center collaboration involving a large cohort of patients.

## 1. Introduction

Radiotherapy prevails in routine clinical practice and plays a cornerstone role in anti-cancer therapy. Imaging, an indispensable part of radiotherapy, aids in delineation of the targeted tumor sub-volume for accurate determination of a uniform therapeutic dose or non-homogeneous dose distribution in the target area. Moreover, advanced clinical imaging tools, such as magnetic resonance imaging (MRI) or positron emission tomography (PET), have been integrated into a radiation system to provide detailed information on anatomy and response of patients before and during radiotherapy 1, 2. Meanwhile, multiparametric MRI (mpMRI), various tracer-based nuclear medicine imaging techniques, and other imaging modalities have been implemented for post-radiotherapy assessment. However, current clinical imaging guidelines 3, 4, such as Response Evaluation Criteria in Solid tumours (RECIST), and RECIST for intratumoral therapies (itRECIST) and immunotherapy (iRECIST), fail to meet the requirements of therapy-specific and timely evaluation of routine and/or emerged combinational radiotherapies (i.e., radio-immunotherapy) 5, 6, whose response patterns may vary from previously well-established one. In this context, novel advanced imaging tools in three main steps in combinational radiotherapies, namely radiotherapy planning, patient stratification, and response/toxicity assessment, are in urgent need 7.

Biomarker-driven molecular imaging has been explored to address these pressing clinical needs. Till now, several radiotherapy-related biomarkers have been identified, including tumor-specific biomarkers for targeted radiotherapy and patient stratification (e.g., prostate-specific membrane antigen (PSMA) and hypoxia) 8, 9, radiotherapy-derived biomarkers for assessment/prediction of tumor response and normal tissue toxicities (e.g., reactive oxygen species (ROS) and caspase-3) 10, 11, and imaging features extracted from functional images (e.g., standard uptake value (SUV) and multiple MRI parameters) 12, 13. The medical research or clinical value of some of these biomarkers has been partially validated or validated in many prospective or retrospective clinical investigations, whereas other specific biomarkers for improving clinical decision-making processes remain to be unveiled, validated, and established. Encouragingly, molecular imaging probes, particularly nanoprobes with a high level of modifiability and multiple imaging modalities 14-17, have been developed to monitor the tumor microenvironment before, during, and after radiation-based therapies in a manner of biomarker-driven activatable imaging. In this context, these imaging probes could be tuned for targeting well-established biomarkers with biological significance, thus offering dynamic and spatiotemporal information for personalized precision therapy to achieve great clinical benefits for cancer patients.

Herein, radiotherapy-specific or its derived imaging biomarkers are surveyed in Chapter 2. Molecular imaging probes for these biomarkers have been developed for imaging-guided/assisted radiotherapy (radiotherapy planning), patient stratification, response assessment, and toxicity/resistance prediction (Chapter 3). Reflection and future perspectives of this biomarker-based molecular imaging for precision radiotherapy are provided in Chapter 4.

## 2. Molecular imaging and imaging biomarkers for cancer radiotherapy

### 2.1. Molecular imaging in radiation oncology

Beyond providing anatomic information, molecular imaging aims to provide fundamental insights/understandings into the pathophysiological process at the molecular and cellular levels under various diseases or therapies-induced changes, thus helping with clinical decision-making. In the context of radiation oncology, molecular imaging with improved specificity and sensitivity plays an important role in radiotherapy planning (e.g., diagnosis and staging, target definition, and image-guided radiotherapy before dose delivery) and early/late-stage treatment response assessment.

In current clinical practice, PET/CT with improved spatial and contrast resolutions and multiparametric MRI are the main workforce, while in preclinical studies, optical imaging, particularly photoacoustic imaging and near-infrared imaging, has emerged. These advanced imaging modalities have been devoted to detecting the minor or significant changes of biomarkers associated with cancer or the radiotherapy-involved pathophysiological process, including but not limited to receptors, cell adhesion molecules, hypoxia, apoptosis, and angiogenesis.

### 2.2. Overview of imaging biomarkers

Broadly speaking, molecular, histologic, radiographic, or physiologic characteristics are examples of biomarkers 18. The multifaceted types of biomarkers have different definitions and classifications 19. In this review, we define their types in radiotherapy (external beam radiation therapy (EBRT), brachytherapy, and radiopharmaceutical therapy (RPT)), varying from diagnostics, prognostics, prediction, and toxicity assessment. To note, biomarkers associated with radiotherapy for indicating biological changes in molecules/proteins, intracellular organelles, cells, and tissues/organs, will be explored and discussed in this review 20. Clinically-investigated biomarkers, such as radiological findings (diametric expansion rate) 21, early metabolic response 22, and imaging parameters 23, will also be briefly mentioned. Additionally, we will specifically discuss the biomarkers that could be employed to develop non-invasive imaging techniques and spatiotemporally assesses tumor lesions (one or more sites) *in vivo* in this review article. Biomarkers that are associated with radiotherapy but cannot be imaged 24, including transcriptomic biomarkers, human papillomavirus (HPV), gene profiles, and extracellular vesicles, are beyond the scope of this review.

### 2.3. Clinically-investigated and emerging imaging biomarkers

**Figure 1:**
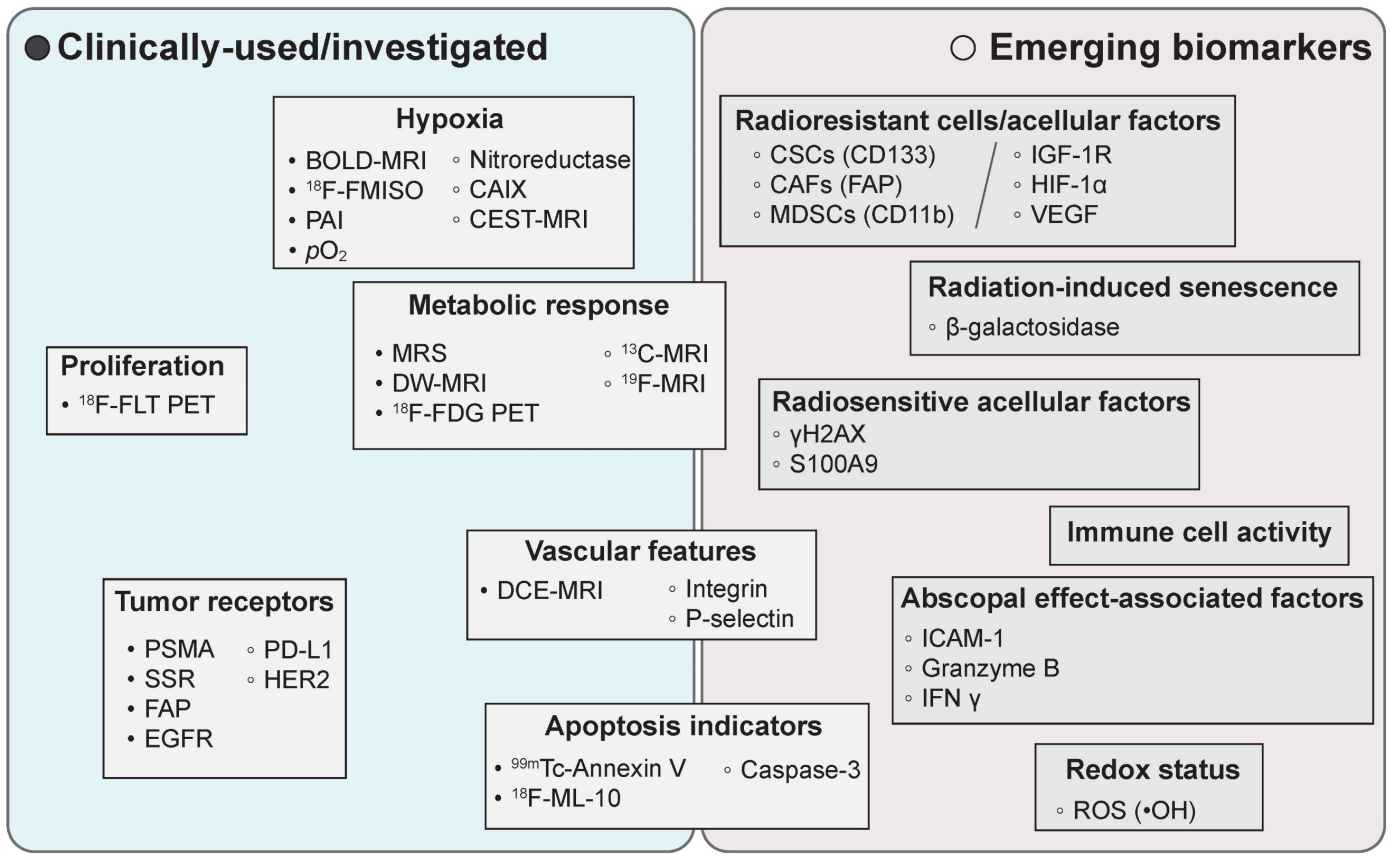
Representative clinically-used/investigated and emerging imaging biomarkers in radiation oncology. BOLD: blood oxygen level dependent; MRI: magnetic resonance imaging; FMISO: fluoromisonidazole; PAI: photoacoustic imaging; CAIX: carbonic anhydrase IX; CEST: chemical exchange saturation transfer; ^18^F-FLT: 3′-deoxy-3′-([^18^F]Fluoro)-fluorothymidine; PET: positron emission tomography; MRS: magnetic resonance spectroscopy; DW-MRI: diffusion-weighted MRI; ^18^F-FDG: 2-[^18^F]fluoro-2-deoxy-D-glucose; PSMA: prostate-specific membrane antigen; SSR: somatostatin receptor; FAP: fibroblast activation protein; EGFR: epidermal growth factor receptor; PD-L1: programmed death-ligand 1; HER2: human epidermal growth factor receptor 2; DCE: dynamic contrast-enhanced; CSCs: cancer stem cells; CAFs: cancer-associated fibroblasts; MDSCs: myeloid-derived suppressor cells; IGF-1R: insulin-like growth factor 1 receptor; VEGF: vascular endothelial growth factor; ICAM-1: intercellular cell adhesion molecule-1; IFNγ: interferon-γ; ROS: reactive oxygen species.

In routine clinical practices, PET and/or MR imaging of hypoxia and metabolic responses prevail in radiotherapy planning and treatment assessment, respectively 23, 25, 26. Moreover, several promising imaging biomarkers and novel imaging techniques have been emerged in pre-clinical studies (**Figure 1**).

***Hypoxia.*** Intratumoral oxygen partial pressure (*p*O_2_) and hypoxia-induced pathophysiological changes (e.g., upregulated expression of nitroreductase) are typical biomarkers for imaging hypoxia 9. Imaging methods for these biomarkers are summarized in **Table 1**. In clinical practices, validated hypoxia measurements have been realized through diagnostic targeted PET probes and MRI techniques, whereas emerged imaging techniques, such as MRI-chemical exchange saturation transfer (CEST) pH imaging and photoacoustic imaging have recently gained popularity for imaging and grading hypoxia 27.

***Metabolic responses.*** Functional multiparametric magnetic resonance imaging (mpMRI) and 2-[^18^F]fluoro-2-deoxy-D-glucose (^18^F-FDG) PET/CT are two major methods to detect early metabolic responses after radiotherapy 39, 40. These imaging results as early predictors, such as apparent diffusion coefficient (ADC), magnetic resonance spectroscopy (MRS), standardized uptake value (SUV) and total lesion glycolysis (TLG), have been frequently explored in radiotherapy for a variety of tumor indications (e.g., esophageal cancer, soft tissue sarcoma) 41, 42. Furthermore, these imaging results in radiotherapy have been reported to be correlated with end points such as recurrence-free survival (RFS) or overall survival (OS) in several prospective/retrospective studies 43-45. For instance, an increased value in the ADC was reported to has a positive correlation with better local control and progression-free survival for mid-RT of head and neck cancer in one prospective study involving 81 patients with a median follow-up of 31 months 43, as well as a declined ADC value after radiotherapy was related with an increased chance of clinical recurrence in a multicenter retrospective analysis of 229 prostate cancer patients 44. In a prospective study involving 62 patients with HPV-related oropharyngeal cancer, SUV, an early PET parameter after 2 weeks of definitive RT (~ 20 Gy), was found to be correlated with RFS and OS 45. In addition, emerged imaging techniques, such as hyperpolarized ^13^C MRI, have been reported to aid in accelerating early detection of metabolic responses (e.g., pyruvate metabolism) as early as 1 day after radiotherapy 46. However, ^18^F-FDG PET/CT lacks specificity, thus it is very challenging to differentiate glucose changes in the brain and liver due to inflammation or radiotherapy. In addition, the ^18^F-FDG uptake rate is only slightly elevated in aggressive subtypes of prostate cancer and neuroendocrine tumors. Other clinically available nuclear imaging probes, e.g., [^18^F]FIMP, have been explored for early-phase assessment of radiotherapeutic responses 47.

***Tumor-associated surface receptors****.* The prostate specific membrane antigen (PSMA) highly expressed on prostate cancer cells and the majority of neovasculature of most solid tumors, and the somatostatin receptor (SSR) highly expressed on neuroendocrine tumors have been successful biomarkers in clinic practices for assisting in both EBRT and RPT in the terms of treatment planning and dosimetry guidance 8, 48. For instance, ^68^Ga-PSMA11 and ^68^Ga-DOTATATE PET probes have been routinely used in the companion diagnosis to adjust the therapeutic dose of ^177^Lu-PSMA617 and ^177^Lu-DOTATATE for patients with prostate cancer or neuroendocrine tumors, respectively. Furthermore, in a recent report, an early change of total lesion PSMA (TLP) (*p* = 0.002) had been found to outperform the serum PSA-based response (*p* = 0.515) in predicting overall survival in 66 metastatic castration-resistant prostate cancer patients treated with ^177^Lu-PSMA617 48. Some other receptors (e.g., fibroblast activation protein (FAP), epidermal growth-factor receptor (EGFR), and integrin α_v_β_3_) with the similar function have also been experimented and trialed 49, 50.

***Radioresistant cell types.*** The density of cancer stem cells (CSCs) (CD133) 51, CSCs-mediated repopulation with increased epidermal growth factor receptor (EGFR) expression 52, the postradiotherapy cancer stemness 53, cancer-associated fibroblast heterogeneity 49, 50, and myeloid-derived suppressor cells and neutrophils are risk factors for radioresistance 54. Imaging probes, such as αCD133-CF770 and [^89^Zr]Zr-DFO-αCD133 have been employed to image CSCs in small cell lung cancer (SCLC) 55. In addition, ^89^Zr-labeled PEGylated anti-CD11b VHHs (antigen binding fragment of heavy chain only antibodies) and [^89^Zr]anti-CD11b antibody (clone M1/70) were reported for immune-PET imaging of the myeloid compartment in colorectal cancer or glioblastoma, respectively 56, 57.

***Radioresistant acellular factors.*** Several biological factors have been identified to induce radioresistance, including DNA repair mechanisms (X-ray repair cross complementing family) 58, tyrosine kinases (insulin-like growth factor 1 receptor (IGF-1R)) 59, metabolic factors (hypoxia-inducible factor 1α, nuclear factor erythroid 2-related factor 2, cyclooxygenase-2), angiogenic regulators (vascular endothelial growth factor (VEGF), osteopontin, interleukin-6) 60, and other factors (NAPDH oxidase 61, galectin-1 62, tissue factor (F3) 63). For their corresponding imaging probes, ^111^In-DOTA-Z_IGF1R:4551_ was reported to image IGF-1R in DU-145 prostate cancer 64; and bevacizumab was designed as a VEGF-A-targeting agent. Its derived imaging probes, including bevacizumab-800CW and gadolinium-diethylenetriaminepentaacetic acid-human serum albumin@indocyanine green-Bevacizumab, have been used in a phase 1 clinical trial to fluorescently visualize soft-tissue sarcomas 65, and guide breast cancer surgery and enhance radiotherapy, respectively 66.

***Radiation therapy-induced senescence (TIS).*** TIS in tumor cells and T cells could be a contributing factor for radioresistance 67, 68. β-galactosidase could be used to probe the senescent status in these cells 69. For instance, a FL/PA bimodal probe, Gal-HCy-Biotin, and a NIR FL/MRI bimodal probe, Gal-Cy-Gd-1, have been reported for *in vivo* imaging of the β-galactosidase activity in tumors 67, 70. In addition, radiation-induced endothelial senescence with a hallmark of the altered interleukin-1 signaling pathway has been found to be associated with lung tissue injury 71.

***Radiosensitive factors.*** Protein biomarkers in human brain metastasis (e.g., S100A9) 72 and those involved in DNA damage response signaling pathways, such as residual γH2AX foci 73, can be used for in-vivo measurements of the intrinsic radiosensitivity level of tumor cells by employing corresponding imaging probes (for instance, [^111^In]In-anti-γH2AX-TAT or [^89^Zr]Zr-DFO-anti-γH2AX-TAT for measuring γH2AX foci).

***Abscopal effect-associated factors****.* The abscopal effect, a phenomenon of the remission of nonirradiated or metastatic lesions beyond the radiation field, occurs infrequently during radiotherapy and it can be attributed to radiotherapy-induced systematically antitumor immune response. Noninvasive imaging of intracellular adhesion molecule-1 (ICAM-1) and granzyme B, using a ^89^Zr-DFO-αICAM-1/Fab PET probe and a DyLight 800- αICAM-1/Fab NIRF probe or a [^68^Ga]-NOTA-GZP PET probe, respectively, has been employed for *in vivo* evaluation of the abscopal effect 74, 75. Other upregulated factors, such as pro-inflammatory cytokine IFN γ, NKG2D ligand, and FAS receptor, may be potential imaging targets 76, 77. However, the abscopal effect is not fully-understood yet and it often has poor reproducibility 78-81. Recent reports have revealed that the blockade of the CD47/SIRPα axis would elicit a macrophage-mediated abscopal effect of radiotherapy and improve abscopal responses 82-84. To note, the immunomodulatory effect may be indirectly assessed by the absolute lymphocyte counts 85 or the number of tumor-infiltrated immune effector cells.

***Redox status.*** The radiotherapy efficacy is partially dependent on the redox potential in the tumor area, namely, the amount of generated hydroxyl radicals and other reactive oxygen species (ROS) after their interaction with antioxidants 86, 87. Furthermore, concerns have been raised that antioxidants may promote cancer progression and metastasis 88, indirectly indicating the essential role of ROS in antitumor effects. In this context, the redox potential may be a promising predictive factor in response to conventional radiotherapy or radiosensitizers-enhanced radiotherapy 86. For instance, *in vivo* dynamic nuclear polarization-MRI using a redox-sensitive carbamoyl-PROXYL probe has been explored for spatiotemporal evaluation of the redox status after radiotherapy 87.

***Apoptosis indicators.*** Tumor apoptosis-associated markers, such as annexin V and caspase-3/7, also act as an initial indicator of radiotherapy efficacy. Based on these biological markers, a number of radiotracers, including ^99m^Tc-Annexin V, ^18^F-ML-10, ^18^F-CP18, and ^18^F-ICMT-11, have been developed to imaging apoptotic cells 89. Moreover, in preclinical studies, caspase-3-responsive ratiometric photoacoustic imaging nanoprobes can be utilized for quantitative assessment of the applied radiation dose 90. However, there is a debate on the role of caspase-3, and it has been argued that caspase may play a role in the cell death-induced tumor repopulation pathway during radiotherapy 91.

***Tumor vascular-related indicators****.* Quantitative tumor-associated vasculature features, including vessel curvature, torsion, and organizational heterogeneity, have been exploited as an imaging biomarker for tumor response in multiple tumor types 92, 93 with the aid of imaging modalities, such as dynamic contrast-enhanced MRI. In addition, decreased tumor blood flow rates (e.g., more than 20%) and receptor expression (e.g., P-selectin) levels have been found to be positively correlated with therapeutic outcomes 94, 95.

## 3. Biomarker-driven molecular imaging probes in radiotherapy workflow

### 3.1. Strengths and roles of imaging probes in radiotherapy workflow

Imaging probes, comprised of small-molecules and nanoformulations, have unique strengths in their application in imaging 14-16, 96, including their inherent imaging properties, surface modifiability, and shape/size manipulability, thus, they have gained increasing interest in radiotherapy workflow. Strengths of applying state-of-the-art imaging probes before, during, and after radiotherapy are briefly listed below: a) Biomarker-specific single/dual/multiple-modality imaging. With the aid of elegant design of probes, such as biomarker-targeted or activated strategies and the use of bifunctional chelators, single/dual/multiple-modality imaging with great sensitivity/tumor penetration, sharp resolution, and enhanced signal-to-noise ratios could be realized 97-99; b) Radiation-responsive imaging probes, such as a phenylalanine and tryptophan-based amino acid nanosensor gel and a gold-containing gel nanosensor 100-102, have been employed for radiation dosimetry in EBRT and/or RPT to quantitatively assess radiation exposure doses; c) X-ray-activated imaging probes, such as perovskite nanocrystal scintillators 103, organic phosphorescent scintillators 104, and probes with the properties of Cerenkov and radioluminescent light enhancement 105, 106, as well as organic luminophores with the ability of radio afterglow imaging 107, have been explored to supplement the contemporary cone beam CT-guided radiotherapy; and d) Probe-mediated immune cell-tracking for monitoring radiotherapy-induced immune response, including the distribution and activity of immune cells. Probe hitchhiking strategies or other direct/indirect cell labelling strategies aid in this tracking procedure 75, 108, 109.

**Figure 2:**
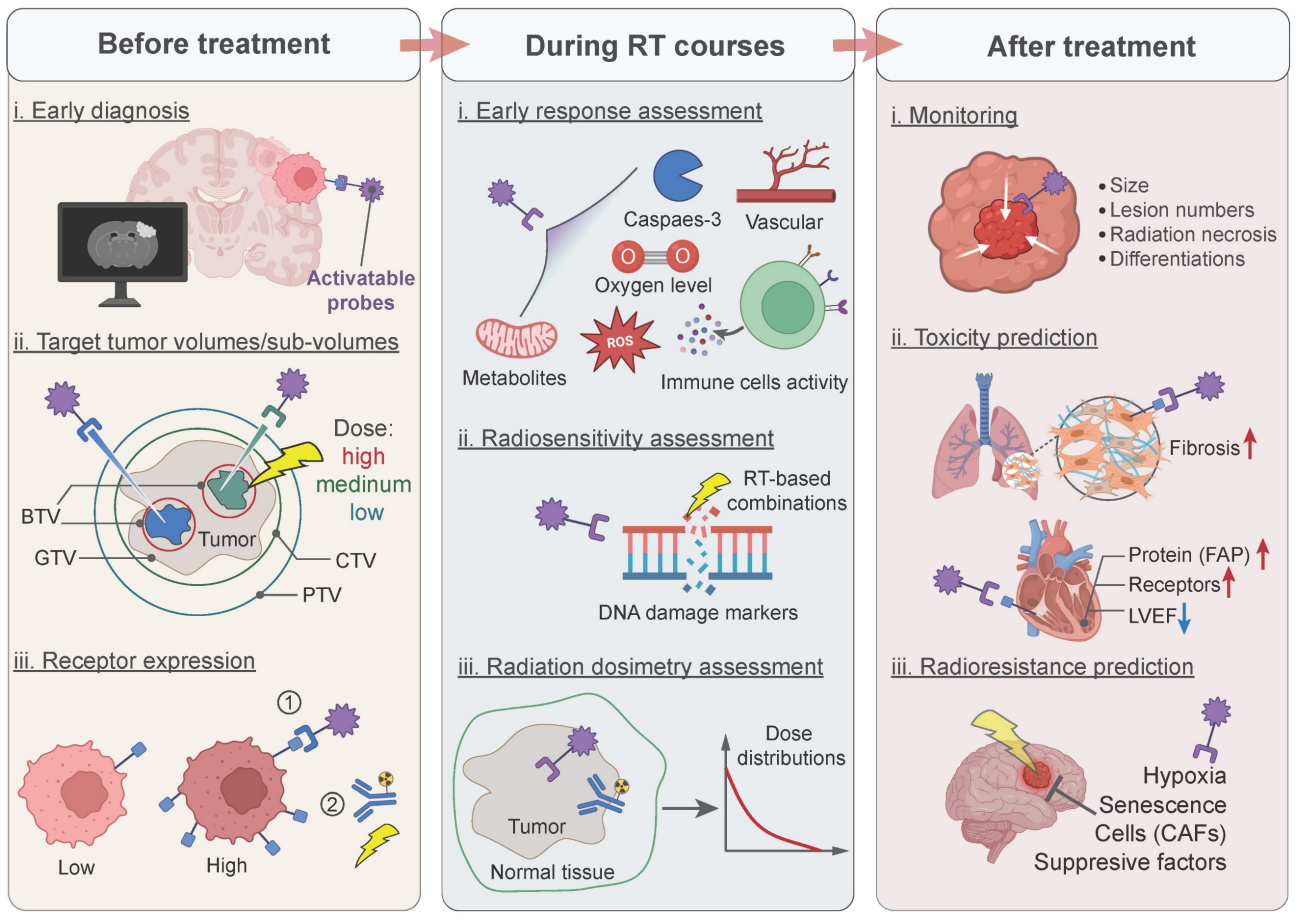
Application of activatable imaging probes in radiotherapy workflow. Before treatment: (i) diagnosing early-stage lesions; (ii) imaging different tumor sub-volumes for dose-escalation; (iii) monitoring the receptor expression for the selection of treatment methods. During radiotherapy courses: (i) assessing early tumor response; (ii) measuring the radiosensitivity index of radiotherapy; (iii) determining radiation dosimetry for optimizing treatment regimes. After treatment: (i) monitoring tumor progression and differentiating imaging results; (ii) predicting radiotherapy-induced toxicity for early interventions; (iii) predicting radioresistance for re-scheduling or changing treatment methods. BTV: biological target volume; GTV: gross tumor target volume; CTV: clinical target volume; PTV: planning target volume; FAP: fibroblast activation protein; LVEF: left ventricular ejection fraction; CAFs: cancer-associated fibroblasts.

**Figure 3:**
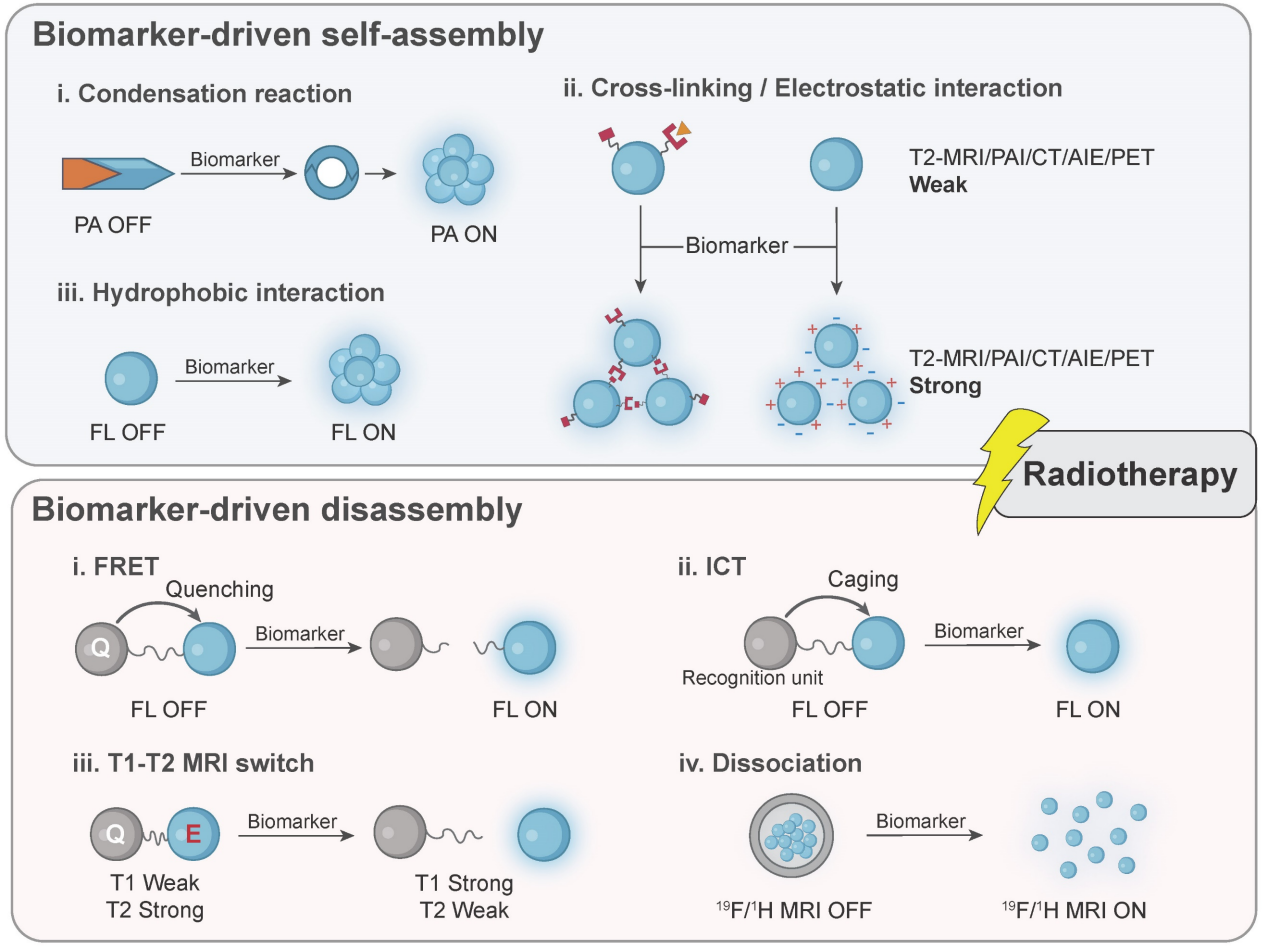
Activatable mechanisms of biomarker-driven imaging probes are divided into two major types: biomarker-driven self-assembly and biomarker-driven disassembly. In situ modification of probes is not illustrated in this scheme. PAI: photoacoustic imaging; MRI: magnetic resonance imaging; CT: computed tomography; AIE: aggregation-induced emission; PET: positron emission tomography; FL: fluorescence; FRET: fluorescence resonance energy transfer; ICT: intramolecular charge transfer.

Taken together, imaging probes, particularly nanoprobes, bring promising and substantial benefits in radiotherapy by broadening the imaging targets and achieving precision therapy through interaction with patient-specific biomarkers. Specific design of them in the workflow of radiotherapy is illustrated in **Figure 2**. Mechanically, biomarker-responsive behaviors of imaging probes have been summarized in **Figure 3**.

### 3.2. Radiotherapy planning

In the current clinical setting, cone-beam computed tomography (CBCT)-guided linear accelerator radiation systems and two MRI-LINAC systems (0.35 T and 1.5 T) have been employed to radiotherapy planning and radiation delivery 110. During biological image-guided adaptive radiotherapy, imaging biomarkers (e.g., markers for amino acids, phospholipid metabolism, peptides, cellular proliferation, hypoxia, and enzymatic activity) are essential to determine non-homogenous dose distribution in segmenting tumors for dose painting 111. In this context, diagnostic biomarker-driven molecular imaging can accurately determine the gross tumor volume (GTV) and reduce the planning target volume (PTV) margin to improve tumor control probability or spare organs at risk (OARs) 112. For instance, images obtained from biomarker-specific PET tracers, such as ^68^Ga-DOTA-FAPI, ^68^Ga-DOTATATE, and EGFR-targeted ^89^Zr-Pantitumumab, could be useful for offline delineation of the GTV (e.g., intracranial meningiomas) 113, 114. It has been revealed from a few clinical trials that greater clinical benefits, for instance, improvements in the failure-free survival 115, the median survival time 116, and the treatment tolerance 117, have been obtained by biological imaging-guided radiotherapy compared to the conventional imaging-guided one 118.

Accurate detection and delineation of GTV play a critical role in the determination of dose escalation or boosting. Tumor-specific biomarkers are often employed as imaging targets in this case, while these imaging biomarkers have to be carefully compared and verified. For instance, a fibroblast activation protein (FAP)-targeted ligand and a prostate-specific membrane antigen (PSMA)-targeted ligand were attached to the surface of core maghemite nanoparticles, respectively, and these nanoprobes were applied in MRI of orthotopic LNCaP prostate tumors. An about 15% improvement in the tumor contrast enhancement and a 1.5-fold increase in the R_2_ values of the tumor periphery were seen in the group treated with FAP-targeted nanoprobes in comparison with the group with PSMA-targeted nanoprobes 119.

**Figure 4:**
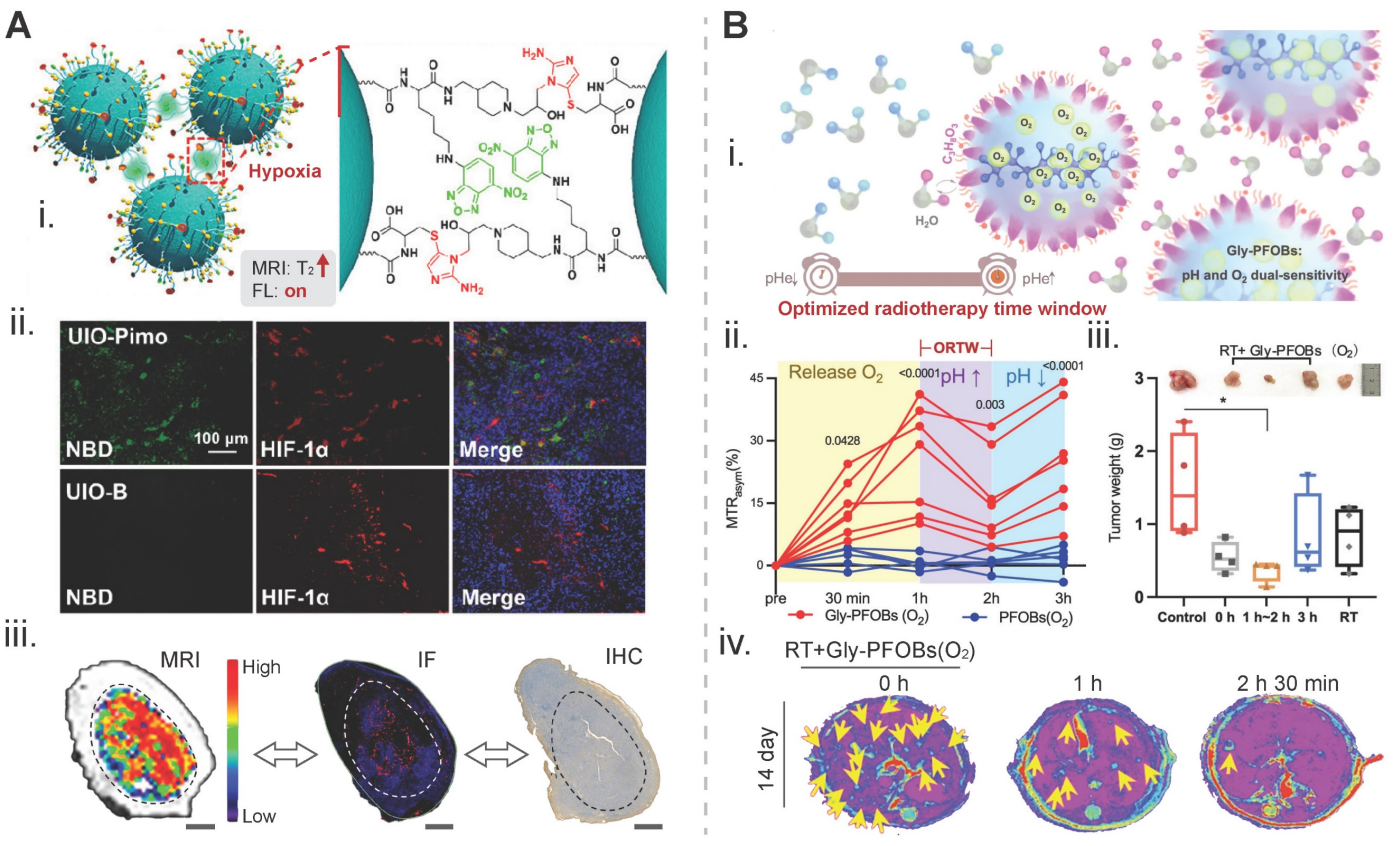
Biomarker-driven imaging probes for radiotherapy planning. A) Hypoxia-triggered self-assembly of an iron-oxide-based MRI/FL dual-mode nanoprobe, termed as UIO-Pimo, for delineation of the targeted tumor area: (i) design principles for imaging signal amplification; (ii) fluorescent images of tumor tissue slices. Red signal from HIF-1α indicates the hypoxia degree and the green signal from NBD represents the fluorescence of the activatable UIO-Pimo nanoprobe and a non-activatable UIO-B nanoprobe; (iii) the distribution of hypoxic areas within a tumor displayed via the nanoprobe-participated MRI difference value method. IF from HIF-1α, and IHC from commercial hypoxia indicator pimonidazole. Adapted with permission 121. Copyright 2021 American Chemical Society. B) A pH/oxygen-activatable ^19^F/^1^H dual-mode nanoprobe for determining an optimal radiotherapeutic window: (i) scheme for the design of the nanoprobe; (ii) dynamic CEST signal changes in a NCI-H460 lung tumor area after injection of Gly-PFOB(O_2_) or PFOB(O_2_); (iii) tumor weights of each group on day 14 post-treatment. The time on the x axis indicats the duration of radiation treatment after injection of Gly-PFOB (O_2_); (iv) T2WI MRI images of the liver in each treatment group, i.e., Gly-PFOB(O_2_) + RT, Gly-PFOB(O_2_) + RT at 1 h post injection of nanoprobe, and Gly-PFOB(O_2_) + RT at 2 h 30 min post injection on day 14 post-treatment. Yellow arrows indicate liver metastasis. Adapted with permission 32. Copyright 2023. Springer Nature. CC BY 4.0 (https://creativecommons.org/licenses/by/4.0/). MRI: magnetic resonance imaging; FL: fluorescence; HIF-1α: hypoxia-inducible factor-1 alpha; IF: immunofluorescence; IHC: immunohistochemistry.

Continuous efforts have been devoted to visualizing the hypoxic tumor sub-volume in modern precision radiotherapy planning, and the design strategy for probes is predominantly based on hypoxia-driven aggregation. A caspase-3 and GSH dual-responsive nanoprobe, consisting of a gold nanoparticle core and a coating containing a DEVD peptide-conjugated AIE molecule INT20, was constructed to realize an optical imaging-guided radiotherapy. The imaging enhancement was achieved through hypoxia-triggered aggregation of INT20 120. In one study, nitroimidazole derivatives as a hypoxia-sensitive ligand and cysteine in a certain ratio were conjugated to the surface of ultrasmall iron oxide nanoparticles, and an aggregation-responsive fluorescence dye, 4-chloro-7-nitro-2,1,3-benzoxadiazole (NBD), was incorporated into these nanoparticles. This dual-mode probe self-assembled via an intermolecular cross-linking mechanism in a hypoxia condition. Strong green fluorescence emitted from responsive NBD in this probe was seen in the 4T1 tumor tissue slices obtained from a hypoxic region-containing tumor with a volume of 1,000 cm^3^ which was predominantly overlapped with a hypoxia marker, HIF-1α. This hypoxia-responsive probe also exhibited a 3.69-fold imaging signal enhancement in T2-weighted MRI (**Figure 4A**) 121. In another study, surface coating of hypoxia-sensitive 2-(2-nitro-1*H*-imidazol-1-yl)ethanamine (NIE) and thiol compounds was applied to gold nanogaps. TEM images clearly indicated distinctive aggregation of nanoparticles when the incubation oxygen level dropped from 21% or 10%, to 1%. In addition, a 4.5-fold increase in the photoacoustic intensity in the in-vivo tumor area was credited to hypoxia-induced aggregation of probes, interestingly, an improvement in the radiotherapeutic efficacy by these gold nanoparticles suggested that they could be radiosensitizers 122.

Determination and utilization of the radiotherapy time window, such as the biological window of FLASH radiotherapy (a dose rate > 40 Gy/s), are essential for improving the therapy efficacy 123. A pH and oxygen dual-sensitive ^19^F/^1^H CEST nanoprobe, Gly-PFOBs (glycerol-weighted and perfluorooctylbromide-based), was developed for imaging-guided lung cancer radiotherapy (**Figure 4B**). The period from 1 h 30 min to 2 h 30 min with a distinct feature of an increased pH was determined to be the optimized radiotherapy time window in an H209 SCLC liver metastasis model 32. In addition, accurate assessment of the time to reach a peak for tumor accumulation of metal theranostic radiosensitizers (e.g., HfO_2_, AGuIX, and iron oxide NPs) using corresponding imaging techniques is critical for amplifying local radiation dose distribution and strengthening tumor cell damage after radiotherapy 124-126.

Overall, biomarkers-targeted imaging probes with high accuracy and specificity can be used to facilitate delineation of the biological-active tumor area, determine an optimal treatment window, and visualize the entire tumor tissue and affected lymph nodes, which will contribute to optimized radiotherapy planning 127. Other radiotherapy guidance techniques using optical imaging are still in the proof-of-concept stage, and more efforts into the development of these techniques are required before they can be translated into clinical use.

### 3.3. Patient stratification

**Figure 5:**
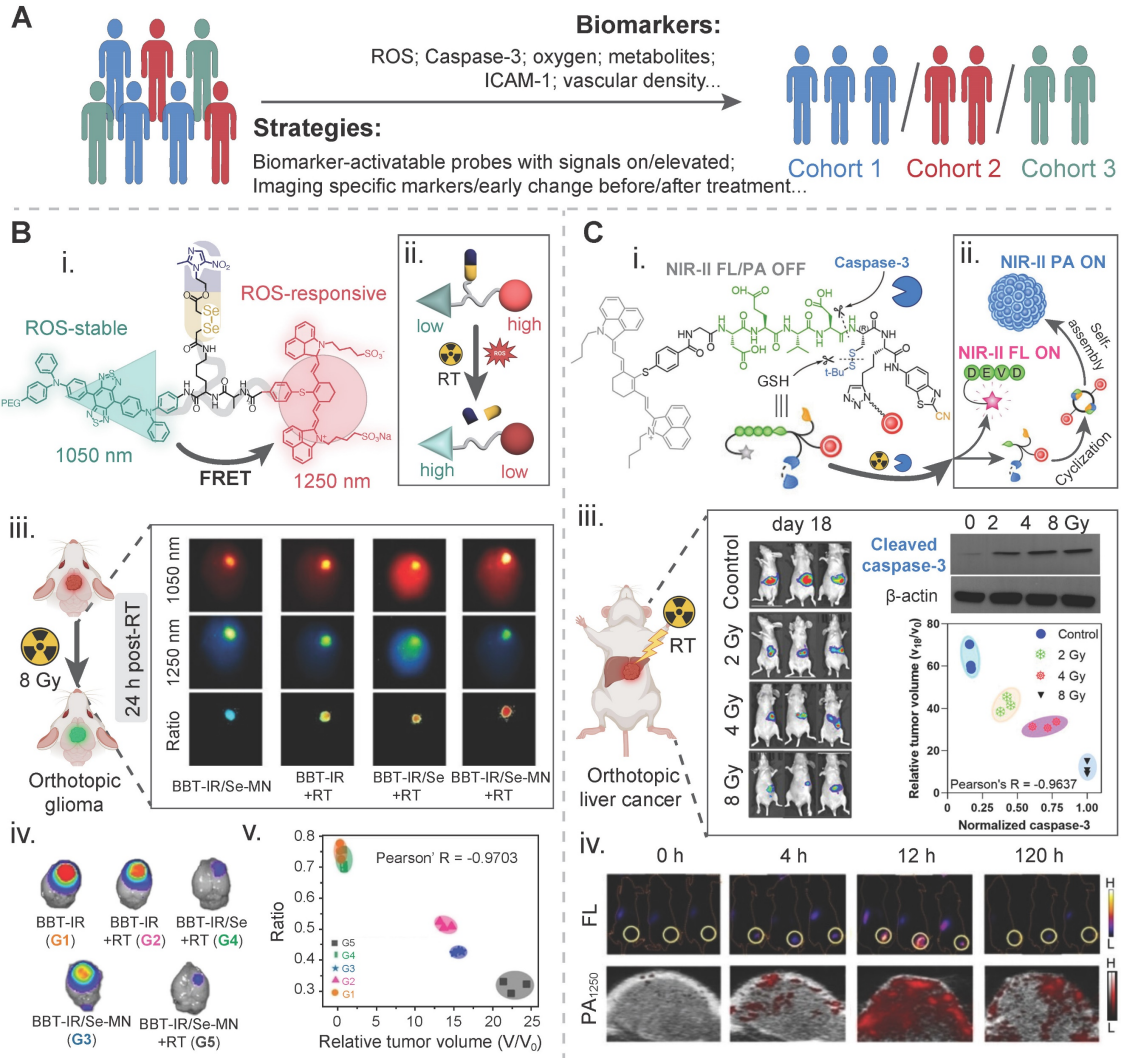
Biomarker-driven imaging probes for patient stratification. A) Illustration of strategies and biomarkers for radiotherapy stratification. B) A tumor reactive oxygen species (ROS)-activatable ratiometric fluorescent probe for reporting the ROS level during radiosensitizer-enhanced radiotherapy: (i) chemical structure and (ii) activatable mechanism of this nanoprobe; (iii) NIR-II FL images of orthotopic glioma-bearing mice in different treatment groups at 24 h post radiotherapy; (iv) bioluminescence images of the brain in the tumor model in different treatment groups on day 16 post radiotherapy. The intensity is an indicator of the tumor mass; (v) correlation between the ratiometric intensity and the relative tumor volume of these above groups on day 15 post radiotherapy. Adapted with permission 136. Copyright 2023 WILEY-VCH GmbH. C) A caspase-3-activatable organic-inorganic hybrid nanoprobe for reporting the caspase-3 level during radiosensitizer-enhanced radiotherapy: (i) chemical structure and (ii) activatable mechanism of this nanoprobe. The red dot for a nanogapped gold nanoparticle; (iii) bioluminescence images of an orthotopic liver cancer model for measuring the tumor mass via the fluorescence intensity, western blotting images of activated caspase-3 in the tumor tissue, and the correlation between the caspase-3 level and the tumor mass in four treatment groups, i.e., the control (0 Gy), 2 Gy, 4 Gy, and 8 Gy; (iv) fluorescence images and photoacoustic images of ectopic xenograft HepG2 tumors under 8 Gy radiation at different time points after injection of the nanoprobe. Adapted with permission 134. Copyright 2022 WILEY-VCH GmbH.

Biomarkers for early risk/benefit susceptibility/stratification could be imaged to distinguish patients with high-response from those with medium- or low-response and determine a patient cohort who is suitable for active surveillance or early intervention 128. In radiopharmaceutical therapies, baseline PET or SPECT scans using biomarker-specific probes provide essential information for selecting patients who are eligible for peptide receptor radionuclide therapy 129, 130. In external beam radiotherapy, molecular imaging of experimentally validated predictive biomarkers or biological features aids in selecting patients who will benefit from specific therapeutic methods. For instance, a multicenter retrospective study using PSMA-PET/CT has indicated that a high mean CT intensity in tumors with a cut-off of 19.7 HU was positively correlated with biochemical recurrence-free survival for patients receiving prostate salvage radiotherapy 131. Overall, the patient stratification strategy helps screening a certain population of cancer patients to receive a beneficial therapy and avoid improper treatment or overtreatment. Oxygen levels, ROS levels, and caspase-3 have constituted typical biomarkers for early tumor stratification in radiotherapy 132-134. Other biomarkers for radiotherapy stratification include the vascular density, metabolites, the intercellular cell adhesion molecule-1 (ICAM-1) (**Figure 5A**).

Photoacoustic imaging has recently emerged as a valuable tool to measure the level of tumor oxygenation 135. To establish the correlation between photoacoustic lifetime-based oxygen images and γ-H2AX-stained histology results in a PDX breast cancer murine model after radiotherapy, an oxygen-sensing G2 polyacrylamide nanoparticle modified with a tumor-targeting F3 peptide, abbreviated as G2-PAA NPs, was injected into the tumor model to monitor the initial oxygen level and map its spatial distribution. After radiotherapy at a dose of 6 Gy was performed on a murine model, immunostaining of γ-H2AX in the resected tumor tissues was conducted. It was found that the local radiotherapy efficacy was positively correlated with the local oxygen level. However, concerns were raised on the calculation method for the structural similarity index with a low spatial resolution of PAI and the misalignment between some of these PAI images and their corresponding histology photos 132.

To enable early detection of radiotherapy-induced ROS, a ROS-responsive ratiometric imaging strategy is often applied because it has the advantage of avoiding false signal. A near-infrared ratiometric fluorescent and photoacoustic dual-model probe that was composed of an •OH-responsive chromophore diene electrochromic material (1-Br-Et), a dye NIR775, and IR1048. Ratiometric FL/PA imaging was performed at twelve hours before and after three treatments on 4T1 tumor-bearing mice: (1) at a dose of 0 Gy, (2) at a dose of 10 Gy, and (3) at a dose of 10 Gy and with tempol as an •OH scavenger to obtain the baseline images and the post-therapy images, respectively. Compared to the baseline images, the normalized ΔFL_780_/ΔFL_1113_ value in the same tumor area treated with 10 Gy was declined by ~2.7-fold, while the normalized ΔPA_755_/ΔPA_905_ value was increased by ~1.4-fold. The changes in these values, particularly the ΔPA_755_/ΔPA_905_ value, could be diminished after the addition of tempol 133. Another ratiometric NIR-II fluorescent molecular probe with a ROS-cleavable diselenide bond, termed BBT-IR/Se-MN, was developed for self-generation of ROS and self-monitoring of the ROS level. In this design, the Förster resonance energy transfer (FRET) between BBT, a ROS-insensitive donor-acceptor-donor fluorophore, and IR, a ROS-sensitive cyanine dye, was disrupted after the reaction of the probe with ROS, leading to a change in the ROS-responsive ratiometric optical signal. The signal intensity at 1050 nm for BBT was increased while the intensity at 1250 nm for IR declined. A negative correlation between the optical intensity ratios and the normalized relative tumor volumes, which corresponded to the early ROS level at 24 h after therapy and the final radiotherapeutic outcome, was established with a Pearson's R-value of -0.9703 in an orthotopic brain murine tumor model (**Figure 5B**) 136.

In vivo quantitative imaging of active caspase-3 within one day after radiation treatment could be used to reveal the early assessment result for the radiotherapy effect. A caspase-3-triggered nanoprobe, AuNNP@DEVD-IR1048, was developed by conjugating a NIR-II FL dye, IR-1048, to the surface of gold nanoparticles via a caspase-3 responsive peptide DEVD. It was found that the relative tumor volume (V_18_/V_0_, 18 and 0 for 18-day and 0-day after treatment) was a function of the normalized caspase-3 activity, as well as the intensity of ΔPA and ΔFL was a function of the relative tumor volume (V_18_/V_0_), respectively. Radiotherapy at three radiation doses of 2, 4, and 8 Gy was performed and non-irradiation one was set as a control, the Pearson's R value between V_18_/V_0_ and normalized caspase-3, ΔPA and V_18_/V_0_, and ΔFL and V_18_/V_0_ was calculated to be -0.9637, -0.9433, and -0.9577, respectively (**Figure 5C**) 134.

Overall, these studies have demonstrated the feasibility of using probes for early-phase imaging biomarkers, and there are correlations between early-phase imaging results and long-term therapeutic outcomes. In this context, patients whose biomarker expression is negative from initial imaging results could be classified into the non-responder group to radiotherapy and other treatment plans with better clinical benefits should be considered for them.

### 3.4. Imaging radiation-induced response: immune cell activity and beyond

To balance patients' risks and benefits, early determination of tumor response is conducive to continuing the current beneficial therapy, switching to an alternative therapy, or developing accurate population models due to tumor heterogeneity. Furthermore, with improved characterizations of the biological response of tumors to treatment methods, particularly, radiotherapy with immunomodulatory effects, advanced and efficacious therapeutic strategies might be developed. In clinical attempts and preclinical studies, several biological imaging techniques, such as diffusion kurtosis imaging 137, multi-parametric MRI 138, and highly sensitive whole-body PET/SPECT or optical imaging 139, have been trialed or tested to assess early tumor response to radiotherapy, concentrating on the level of cellular communities and acellular components.

**Figure 6:**
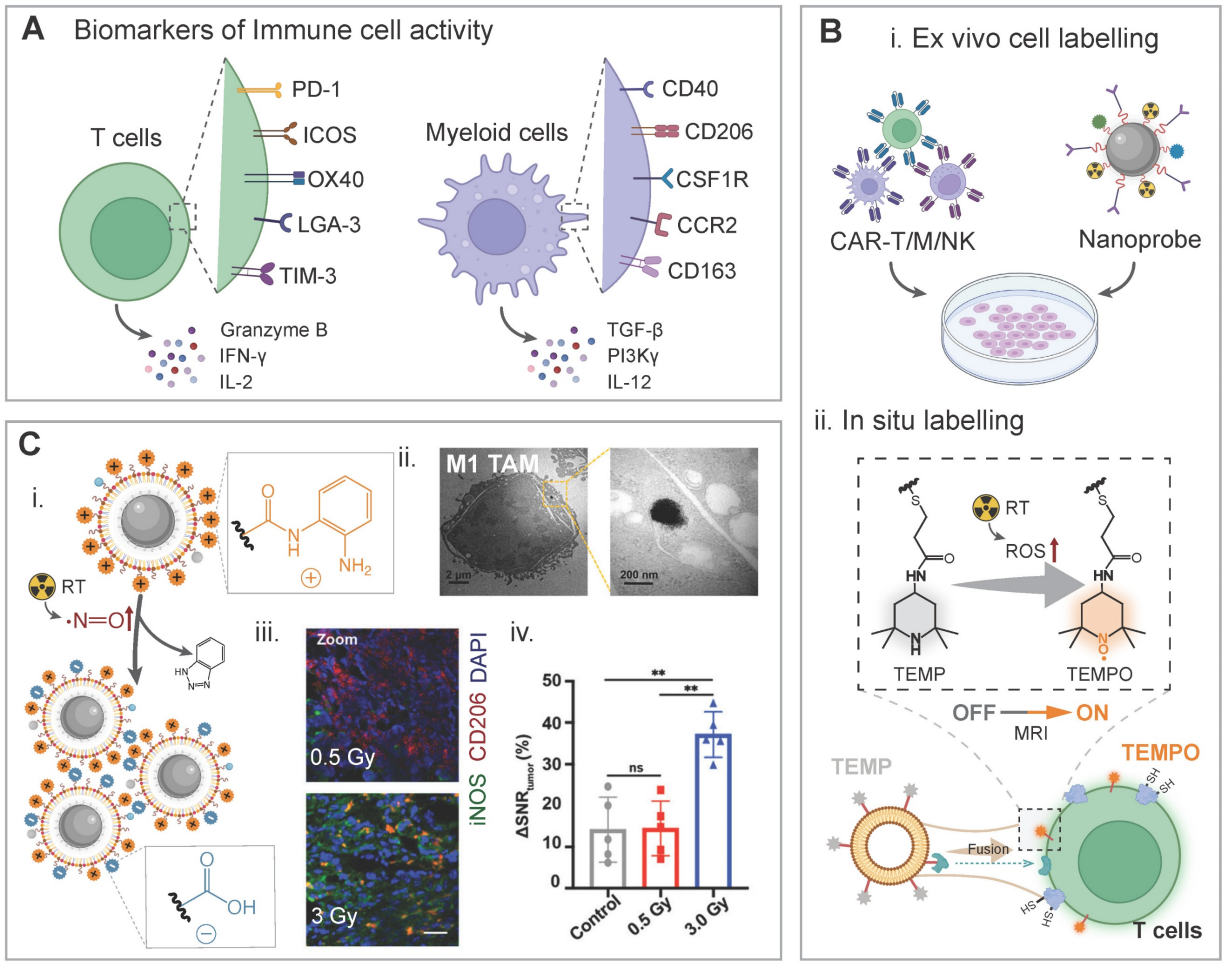
Biomarker-driven imaging probes for immune cells and their activities. A) Schematic illustration of biomarkers for immune cell activities during radiotherapy, including up/down-regulated surface receptors and secreted cytokines. B) Imaging of immune cells can be realized by ex vivo cell labelling and in situ labelling. Imaging-visible nanoprobes were ex vivo labelled with CAR-T/M/NK cells for real-time monitoring of their biodistribution, and T cell-directed liposomes were fused with T cell for in-situ labelling and they were activated when encountering radiation-induced ROS. C) Nitric oxide-triggered self-assembly of the USPIO@OMG nanoprobe for evaluating macrophage polarization during radiotherapy: (i) chemical principle for aggregation of the nanoprobe; (ii) TEM images to confirm the aggregation status of the nanoprobe in M1 macrophages; (iii) CLSM images of tumor slices from two radiation dose-treated groups, red CD206 signal indicates M2 macrophages while the green iNOS signal represents M1 macrophages; (iv) The T2 MRI signal changes in different groups. Adapted with permission 147. Copyright 2023 American Chemical Society.

***Imaging immune cells and their activity.*** Specific surface receptors of immune cells and their secreted cytokines have been reported to be regulated during radiotherapy, and some of them have been correlated with therapeutic outcomes (**Figure 6A**) 86, 140-142. In combination therapies with radiotherapy, imaging targets or biomarkers (e.g., surface receptors or secreted cytokines) for the T cell activity include programmed death-1 (PD-1), inducible T cell co-stimulator (ICOS), OX40, lymphocyte-activation gene 3 (LGA-3), T cell immunoglobulin and mucin domain-3 (TIM-3), granzyme B, interferon-gamma (IFN-γ), and interleukin-2 (IL-2). Biomarkers for the myeloid cells activity include CD40, CD206, colony-stimulating factor 1 receptor (CSF1R), C-C chemokine receptor 2 (CCR2), CD163, transforming growth factor beta (TGF-β), phosphatidylinositol 3-kinase gamma (PI3Kγ), and IL-12. In this context, nanoprobe hitchhike strategies/biomarker-targeted imaging strategies could be employed for imaging immune cells or monitoring their status/activity via a direct/indirect adherence manner (**Figure 6B**). Briefly, imaging probes can be attached onto immune cells or bound to secreted cytokines via receptor-ligand interaction for *in vivo* monitoring, and the biomarker expression, indicated by the intensity of imaging signals, can be correlated with the cell activity. In addition, longitudinal imaging of CAR T cells or other ACT cells via reporter genes or other methods provide additional insights into their biodistribution and tumor-infiltration, and the biological effect of radiotherapy in the era of cancer radio-immunotherapy 143, 144.

Per-fluoro-crown-ether (PFCE) nanoparticle-mediated ^19^F MRI was explored to longitudinally monitor tumor-associated macrophages (TAMs) in glioma-bearing mice after radiotherapy and a radiotherapy recurrent gliomas model. The dynamics of TAM subpopulations were successfully monitored through MRI, while the NP uptake mechanism by different TAM phenotypes during the intervention of radiotherapy remained to be identified 145. In addition to this novel imaging technique, conventional ^1^H MRI and optical imaging approaches have been optimized and tuned for dynamic monitoring of TAMs during radiotherapy. For example, dynamic imaging of TAMs in response to low-dose radiotherapy (5 Gy) via a metabolizable dextran-indocyanine green NIR-II nanoprobe was conducted in SW1990 pancreatic tumor-bearing nude mice. The TAM-specific targeting was realized through the interaction of dextran in this probe with ICAM-3-grabbing nonintegrin-related 1 on TAMs. After three days post X-ray treatment, fluorescence images of the tumor area at different time points (6, 12, and 24 h) post-injection of the probe were recorded. A 2-fold increase in the signal intensity was found in the radiation-treatment group, compared to the non-treatment group and the TAM-depleting group 146. Phenotypic polarization of macrophages during radiotherapy may be indirectly indicated with an elevated NO molecule level in the highly responsive tumor, thus the NO level could be used as a specific biomarker for TAM repolarization from the M2 to M1 phenotype. An ultrasmall paramagnetic iron oxide-based nanoprobe was modified and the aggregation of the nanoprobe could be triggered with an increase in the nitric oxide concentration. Comparing the treatment effects in a 4T1-tumor bearing murine model at three radiation doses (0 Gy, 0.5 Gy, and 3.0 Gy), a similar trend was observed for the ΔSNR_tumor_ in the T1 and T2 settings and the M1/M2 ratios in these groups: a significant change in the imaging contrast and an increase in the M1/M2 ratios in the 3.0 Gy-irradiated group compared to those in the non-treated group and the 0.5 Gy-treated group, while no statistical difference in both the imaging contrast and the M1/M2 ratio between the non-treated group and the 0.5 Gy-treated group (**Figure 6C**) 147.

Other tumor-infiltrating immune cells have been imaged during radiotherapy. A circulating monocyte-hitchhike strategy was developed using a fluorescently labeled theranostic nanoprobe to monitor low-dose RT-facilitated monocyte chemotaxis. Mechanically, this nanoprobe could hitchhike RT-recruited monocytes via the interaction between its surface-modified lipoteichoic acid and the CD14 receptor on monocytes 148. To specifically track tumor-infiltrating leukocytes (TILs), a series of “dual-locked” activatable near-infrared nanoprobes were developed. They were activated upon exposure to both leukocyte biomarkers (caspase-1, granzyme B, or neutrophil elastase) and the tumor biomarkers (aminopeptidase N). The selectivity of these nanoprobes towards specific biomarkers was verified both *in vitro* and *in vivo*. More importantly, these nanoprobes could differentiate subpopulations of TILs, and they could be employed for patient stratification and therapeutic efficacy prediction 149.

***Imaging hypoxia****.* Probes have also been developed to monitor the hypoxic dynamics (magnitude and distribution) to evaluate the level of tumor reoxygenation in the tumor during radiotherapy. It was revealed by a near-infrared phosphorescent nanoprobe, consisting of a fluorescent semiconducting polymer and a palladium complex, that a higher fractional dose (10 Gy × 3 f) was more effective in improving tumor reoxygenation in a Hela tumor (diameter: 8 mm)-bearing nude murine model than a lower dose (2 Gy) 150. Another phosphorescent probe with Cherenkov-excited luminescence imaging properties, Oxyphor PtG4, was developed to image *p*O_2_ distribution during radiotherapy. After analyzing the obtained *p*O_2_ images with various spatial resolutions in the range of 0.2 mm to 5 mm, it was found the hypoxic fractions was decreased by more than 50% after radiotherapy at a dose of 5 Gy × 5 f on MDA-MB-231 or FaDu tumor-bearing murine models, or 8 Gy on MDA-MB-231 tumor models, and accurate imaging was realized by images with a high spatial resolution of 0.2 mm 151.

***Imaging the redox status.*** A radiation-induced ROS-activatable disassembled nanoprobe was constructed from a PEG-PPS-PEG-NH2 polymer, iron oxide nanoparticles (IO NPs), and DOTA-Gd. In this design, radiation-induced ROS-responsive “turn-on” of the T1 signal was achieved through the disassembly of the nanoprobe via the oxidation of hydrophobic thioethers to hydrophilic sulfones and an increased distance between IO NPs as a quencher and DOTA-Gd as an enhancer. A high Pearson's correlation coefficient was observed between changes in the T_1_ signal changes at 24-48 h post-treatment and the frequency of CD4^+^CD8^+^ T cells as an indicator for adaptive immunity (R = 0.9831) or the tumor inhibition rate (R = 0.9308) after radiotherapy. Therefore, this nanoprobe could be employed for early and accurate evaluation of radiotherapeutic treatment outcomes at 24-48 h post-treatment 10.

***Imaging caspase-3.*** A caspase-3-activated probe with “turn-on” fluoro-photoacoustic signal was developed for early evaluation of the external radiotherapy effect. Self-assembly of this probe was triggered under the stimulation of upregulated caspase-3 induced by radiotherapy and significant changes in the fluorescence intensity and the photoacoustic intensity were detected. In an EL4 lymphoma tumor-bearing murine model treated with a range of radiation doses (i.e., 0, 1, 2, 5, 10 Gy), the normalized fluorescent intensities and ΔPA signal intensities were positively correlated with the radiation doses at 3-24 h post *i.v.* injection of the probe. Furthermore, they were negatively correlated with the relative tumor size on day 21 post-radiotherapy with a high Pearson's R-value of -0.8989 and -0.9428, respectively, implying that a stronger signal enhancement at the tumor sites after radiotherapy may be used to predict a smaller tumor size after radiotherapy 152. A PSMA-targeted, ^177^Lu-labeled theranostic probe conjugated with a hemicyanine-based NIR agent via a DEVD peptide cleaved by caspase-3 was prepared for fluorescence imaging to assess the elevated level of internal radiotherapy-induced caspase-3. Statistically, this theranostic probe had a low *K*_i_ value of 4.12 nM and a low *K*_m_ value of 67.62 μM, suggesting it had strong binding affinity to PSMA. The detection of limitation was down to 0.125 U/ml of caspase-3 which was derived from the fluorescence signal. Therefore, it was very sensitive to changes in caspase-3. In a medium PSMA-expressing 22Rv1 prostate cancer model, this probe achieved a 1.79-fold fluorescence contrast enhancement 153.

Taken together, conventional fMRI scanning approaches and other quantitative imaging modalities have already been used to monitor the biological response in the tumor during the treatment procedure for external/internal radiotherapy 125, 154. These quantitative imaging modalities also lay a solid foundation for developing biomarkers-driven molecular imaging with specificity and accuracy. After radiation-associated biomarkers have been experimentally identified or preliminarily validated from gene, RNA, and protein analysis, specific imaging probes targeting these biomarkers could be prepared, and quantitative imaging modalities are then applied to image these molecular biomarkers.

### 3.5. Predicting toxicity potential and resistance

Radiotherapy-induced adverse effects have been reported, including brain injury 155, radiation dermatitis 156, bone loss, radiation-induced intestinal fibrosis (RIF) 157, and radiation-induced acquired therapy resistance. The success or failure of a therapy would be heavily relied on these adverse effects. Conventionally, treatment (e.g., radiation)-induced adverse events are graded according to the Common Terminology Criteria for Adverse Events (CTCAE v.5.0). Imaging techniques based on toxicity- or resistance-associated biomarkers could provide an alternative non-invasive, spatiotemporal, and objective assessment approach to implementing these criteria for assessing radiation-induced adverse effects. Meanwhile, advanced imaging processing tools, particularly machine learning and artificial intelligence, may play a crucial role in predicting treatment failure and RT-induced toxicities 158.

In general, excess ROS generated after radiation accounts for the principal source of radiation-induced toxicity (e.g., acute esophagitis and vasculitis.) 11, 159, 160. Hypoxia, therapy-induced senescence, and resistant cellular types are the predominant contributors for radioresistance. Accurate and timely elucidation of normal tissue injury and tumor tissue radioresistance using these biomarkers-driven imaging probes in an non-invasive and spatiotemporal manner is conducive to risk stratification, early intervention, and re-optimization of treatment plans.

***Radiation-induced lung injury (RILI).*** It has been reported that the majority of radiation pneumonitis occurs within 4-12 weeks after completion of radiation therapy 161, 162. In light of steroids intervention has a good therapeutic effect on radiation pneumonitis at its early phase, the biomarkers for early detection are essential to improve the patient outcomes. An earlier marker, i.e., C-X-C-chemokine-receptor-type-4 (CXCR4), was explored for diagnosis with the aid of a CXCR4-targeted PET probe, [^18^F]AlF-NOTA-QHY-04. A RILI rat model and a large population of 14 patients with radiation pneumonia were included in this study. A remarkably higher peak of the imaging signal was observed in the irradiated lung tissue than the unirradiated one on day 14 post-radiotherapy in rat models, and cancer patients with grade-2 RILI exhibited a significantly higher SUVmax value than those with grade-1 RILI 163. To allow specific evaluation of radiation-induced lung fibrosis, an integrin ανβ6-targeted PET probe, [^18^F]-FBA-A20FMDV2, was used in an imaging study. *S*ix non-small cell lung cancer patients after radiotherapy and six healthy volunteers were included in this study. A higher mean PET uptake of probes was observed in the irradiated lung compared with the healthy control (2.97 vs 1.99, *p* < 0.05). Furthermore, there was a remarkable pharmacodynamic relationship between the uptake level of the integrin-targeted tracer and the radiation dose 164. This preliminary study suggests that integrin ανβ6 could be used as an imaging target for radiation-induced lung fibrosis. Two trials have confirmed the feasibility of using type 1 collagen as a RILI biomarker (NCT04485286 and NCT03535545). In both trials, two distinctive type 1 collagen-targeted probes, a MR probe, EP-3533, and a PET probe, ^68^Ga-GBP8, were applied to image the developed murine model after lung radiation, as well as excised human lung tissues with RILI from six human subjects. The imaging signal intensity was well correlated with the severity degree of progressive fibrosis, indicated by the collagen proportional area in excised tissues, and the RILI area in human subjects 165.

***Radiation-induced myocardial damage.*** One should note that radiation even at a low dose might cause toxicity, such as a risk of heart diseases 166. Coronary artery calcium (CAC) scores 167, serum lipopolysaccharide-binding proteins 168, and the MRI-derived extracellular volume are set as prognostic risk factors for adverse cardiac events after thoracic radiotherapy.

For imaging radiation-induced cardiotoxicity (e.g., cardiac remodeling and fibrosis), several potential targets could be used, such as somatostatin receptors, αvβ3 integrin receptor, fibroblast activation protein (FAP), norepinephrine transporter, mitochondrial membrane potential, and ROS 169-171. In a retrospective study of 32 cancer patients undergoing ^68^Ga-labeled FAPI PET examination, myocardial uptake of these tracers was found to be associated with coronary artery disease (CAD), age, and left ventricular ejection fraction (LVEF), indicating these parameters could be considered in early risk stratification 172. Early detection of radiation-induced myocardial damage using ^18^F-AlF-NOTA-FAPI-04 PET/CT imaging can be realized before a decrease in left ventricular ejection fraction. Specifically, in rat models, the myocardial FAPI uptake was significantly increased at 2^nd^ week and peaked at 5^th^ week post-radiation, whereas the LVEF was dramatically declined at 8^th^ week 173.

Several serum biomarkers and urine biomarkers have been evaluated for early and accurate assessment of radiation-induced toxicity 168, 174, 175, but failed to locate the toxicity origins. To this end, early imaging biomarkers for a specific manifestation are actively screened to achieve monitoring of a spatiotemporal toxicity profile.

***Radioresistance.*** To accurately profile the degree of radioresistance is essential for reaching treatment decisions. As galectins are positively correlated with the hypoxia level, a ^68^Ga-galectracer PET probe, ^68^Ga-NOTA-PEG4-TDGd, was constructed by radiolabeling a thiodigalactoside derivative to explore its prediction capacity of radioresistance. This prediction capacity was confirmed in a 4T1-bearing mice model, and a higher tumor uptake of the galectin-targeted tracer was associated with significantly poorer tumor response to radiotherapy 176. Cancer-associated fibroblasts (CAFs) constitute nearly 90% of the whole tumor volume in epithelial tumors, and they play a crucial role in mediating radio-resistance 177. It is noted that the fibroblast activation protein is a valuable biomarker for CAFs. Prior to the construction of targeted imaging probes for the well-defined biomarker, effective FAPI ligands, including FAPI-46, FAPI-46-F1D, FAPI-46-EB, FAPI-46-Ibu, FAP-2286, have been developed 178. In addition, transforming growth factor β (TGF β), a therapeutically negative factor, could be a targetable imaging marker during radiotherapy. A TGF β neutralizing mAb was radiolabeled with PET radionuclide ^89^Zr to form ^89^Zr-fresolimumab and it was then applied to image radiation-induced TGF β activation. To screen active TGF β expression from tumor slices and the tumor radioactivity from *in vivo* PET-CT images, comparisons were made between the active and latent TGF β status in two cell lines (C19 and B9), as well as between the αvβ8-positive and αvβ8-negative Lewis lung carcinoma (LLC). It was confirmed that ^89^Zr-fresolimumab could actively and specifically image TGF β activation. This probe-mediated PET imaging and four treatments (sham, 1D11 as a TGF β inhibitor, RT, and RT + 1D11) were performed on three distinctive intracranial tumor models, including two murine glioblastomas (GL261 and SB28) and one brain-adapted 4T1 murine breast cancer. To note, 1D11 exhibited a similar survival curve as the sham group, while the combination of RT with 1D11 significantly extended the survival rate compared to other groups. These extended benefits were found to be correlated with the level of TGFβ activity detected by PET imaging 179.

However, these novel potential biomarkers for radiotherapy imaging are identified and validated from multiple cohorts of clinical trials or practices, and their clinical values remain to be verified. These biomarker pools are critical for future implementation of personalized radiotherapy.

## 4. Reflection and future perspective

Five phases are usually involved in the development of biomarkers: preclinical exploratory, clinical assay and validation, retrospective longitudinal monitoring, prospective screening, and cancer control 180. Through these steps, potential biomarkers have been reported in preclinical and clinical trials, while these biomarkers await robust validation and accurate qualification, and only those associated with clinical outcomes could be considered for clinical use. It is expected that biomarker-driven molecular imaging could provide crucial information to make 'go or no-go' decision after identification of treatment failure at an early stage so that side effects could be avoided for patients. Meanwhile, positive outcomes from imaging biomarkers at the early stage of treatment may act as indicators for surrogate clinical endpoints of successful treatment.

Humanized patient-derived xenografts mouse models are preferred for identifying and validating radiotherapy-specific biological or radiological biomarkers 181-183. The accurate tumor-specific anatomic information and dynamic multi-modality/multi-parametric functional images from biomarker-driven probe-mediated molecular imaging could provide a large and reliable training source for the development of advanced imaging processing tools, such as machine learning and artificial intelligence 184. In clinical trials, the imaging evaluation system needs to consider these parameters: the eligibility criteria, endpoints, trial feasibility, as well as interobserver variabilities in the tumor size measurement. In this context, predictive biomarkers instead of prognostic ones could be explored to pre-determine the endpoint of clinical trials 185. In addition, new potent endpoints, for instance, growth modulation index (GMI), could be incorporated into the imaging evaluation system.

Reflection and future perspectives of developing imaging biomarkers and biomarker-driven molecular imaging probes in radiotherapy are provided after we carefully review previous successes and failures of clinical and preclinical studies.

I. Technical and clinical validations of biological imaging biomarkers are currently very active. Unfortunately, the majority of them fail during their translation into a medical research tool, and significantly challenging issues lie in their translation into a decision-making tool, because of their low screening, diagnostic, or predictive valence 23. Thus, at the first beginning, imaging biomarker candidates, particularly molecular biomarkers, should be obtained from a robust procedure of identification and validation using multiple molecular techniques, such as cDNA microarray analysis, clone sequencing, oligonucleotide arrays, reverse-phase protein lysate arrays, and tissue microarrays. In this context, the defined molecular biomarkers and their correlations with clinical benefits can have a solid biological basis. False/negative/positive imaging signal resulting from chemical, biological, or structural changes of biomarkers, especially the enzyme, could be avoided.

II. It is of utmost importance to reveal typical response patterns or biological alternations induced by various radiotherapy modalities/sources and different combinational radiation therapies in cancer patients or clinical samples. These findings can therefore provide the guidelines of predicting long-term clinical outcomes and radiation-induced side effects, and accelerate clinical use of beneficial combinational radiotherapy. Critically, evaluation of these novel molecular imaging biomarkers should be conducted in a larger patient cohort. Additionally, in agreement with one of the recommendations proposed by the Consensus Statement 23, all true-negative, false-negative, and false-positive data obtained from these exploratory studies on either animals or humans should be published or shared in a public database.

III. As radiopharmaceutical therapy (RPT) has been expected to be the mainstream of radiation oncology practice, its differences from external beam radiotherapy (EBRT) in dosing rates and dose distributions may lead to unexpected treatment response and toxicities. The major concern will be patient-specific dosimetry in treatment planning 186. Biomarker-targeted imaging probes could be explored to provide critical information on the receptor phenotype and its expression level, as well as its binding sites, which helps bridging the knowledge gap in revealing the differences between RPT and EBRT. In addition, during translation of preclinical results into clinical trials, one should note that the receptor phenotype and expression can vary significantly between human models and rodent/ murine tumor models before, during, after radiation therapy 187. Pharmacodynamics biomarkers and special imaging effects, such as the tumor sink effect in large-tumor-bearing patients, should also be considered when interpreting imaging results.

IV. Specific and adequate accumulation of probes for imaging radiotherapy in the area of interest with robust imaging enhancements and a great safety profile should be addressed during the design step. In this context, from a chemical structure perspective, the ligands for targeting biomarkers 178, the linkers cleavable by biomarkers 188, 189, the chelators for paramagnetic ions or diagnostic radionuclides 190, and the biomarker-activatable imaging enhancement principles should be considered for the probes 191. The basic *in vivo* working mechanism(s) of a certain probe type, including tumor targeting 192, excretion 193, and signal activation 14, should become the cornerstone for the development of probe-mediated molecular imaging. Novel nanoprobes from the same nanoprobe type could help shortening their development time, while their preclinical and clinical data could continuously and dynamically mature the mechanism(s) of this nanoprobe type.

V. In the roadmap of biomarker-driven imaging probes towards clinical translation, consensus guidelines on their synthesis methods and performance of biological imaging-guided adaptive radiotherapy are essential to reduce the bias or variability between interobservers, inter-institutions or intra-institutions 194. In addition, instrumental contributions are fundamental to improve radiotherapy planning, including synthetic computed tomography for MRI online adaptive planning 195 and optimization of imaging reconstruction algorithms in dosimetry 196. In the era of precision radiotherapy, the use of advanced techniques such as artificial intelligence (AI) and machine learning (ML) is on the rise 197. The radiomics features with quantitative metrics (texture, morphology, and dosimetry) extracted from imaging modalities such as PET or MRI are abundant and they could be explored to assess biological, physiological, metabolic changes before, during and after radiotherapy, such as identifying the T cell exhaustion status 198 and predicting pathological complete response of neoadjuvant radiotherapy 199.

## 5. Concluding remarks

Individual biological targets are the foundation of personalized radiotherapy. Imaging biomarkers at genetic, proteomic, and metabolomic levels can provide informative guidance for radiotherapy interventions. Thus, comprehensive spatiotemporal imaging of molecular biomarkers and quantitative analysis of these radiomics features help achieving better cancer management. Although imaging biomarkers have been widely explored and assessed, evaluations of them has not been conducted in a large patient cohort. Very few biological biomarkers that are well correlated with radiotherapeutic outcomes including clinical benefits and side effects have been identified and verified. Collaborative efforts should be made into building the correlations of imaging biomarkers with biological and pathological changes, tumor growth control degrees, side effects to normal tissues before, during and after radiotherapy or combined radiotherapy. It will be equally important to develop nanoprobes for these imaging biomarkers which can be readily translated from rodent/murine tumor models to human subjects.

## Figures and Tables

**Table 1:** Representative clinically-used or investigated biomarkers for imaging hypoxia.

Biomarkers	Imaging methods	Refs
Hypoxic biological features	2-nitroimidazole compounds-based PET tracers (^18^F-FMISO, ^18^F-FETNIM, and ^18^F-FAZA) or SPECT tracers	28
	^64^Cu-ATSM PET tracer	29
	^99m^Tc-labelled SPECT tracer	30
	^123^I-IAZA SPECT tracer	31
*p*O_2_	^19^F-relaxometry	32
	EPRI oximetry	33
	Fiber-optic oxygen-sensing devices	34
Perfusion	Dynamic contrast enhanced MRI	35
	Computed tomography	36
Hemoglobin-oxygen saturation	R2* (Spin dephasing rates) from MRI BOLD sequence	37
	Photoacoustic imaging	38

PET: positron emission tomography; FMISO: fluoromisonidazole; FETNIM: fluoroerythronitroimidazole; FAZA: fluoroazomycin arabinoside; SPECT: single photon emission computed tomography; Cu-ATSM: Copper (II)-diacetyl-bis(4-methyl-3-thiosemicarbazone); IAZA: iodoazomycin arabinoside; *p*O_2_: intratumoral oxygen partial pressure; EPRI: electron paramagnetic resonance imaging; BOLD: blood-oxygen-level-dependent.

## Abbreviations

ADC: apparent diffusion coefficient
AIE: aggregation-induced emission
BOLD: blood-oxygen-level-dependent
BTV: biological target volume
CAFs: cancer-associated fibroblasts
CEST: chemical exchange saturation transfer
CSCs: cancer stem cells
CT: computed tomography
CTV: clinical target volume
EBRT: external beam radiation therapy
EGFR: epidermal growth factor receptor
EPRI: electron paramagnetic resonance imaging
FAP: fibroblast activation protein
FL: fluorescence
FRET: fluorescence resonance energy transfer
GTV: gross tumor target volume
HPV: human papillomavirus
ICAM-1: intracellular adhesion molecule-1
ICT: intramolecular charge transfer
IGF-1R: insulin-like growth factor 1 receptor
LVEF: left ventricular ejection fraction
MRI: magnetic resonance imaging
mpMRI: multiparametric magnetic resonance imaging
MRS: magnetic resonance spectroscopy
OS: overall survival
PAI: photoacoustic imaging
PET: positron emission tomography
PFS: progression-free survival
*p*O_2_: oxygen partial pressure
RPT: radiopharmaceutical therapy
PSMA: prostate-specific membrane antigen
PTV: planning target volume
RILI: radiation-induced lung injury
RECIST: Response Evaluation Criteria in Solid tumours
ROS: reactive oxygen species
SPECT: single photon emission computed tomography
SUV: standard uptake value
TAMs: tumor-associated macrophages
TGF β: transforming growth factor β
TILs: tumor-infiltrating leukocytes
TLG: total lesion glycolysis
